# Local analgesia in paediatric dentistry: a systematic review of techniques and pharmacologic agents

**DOI:** 10.1007/s40368-017-0302-z

**Published:** 2017-09-14

**Authors:** G. Klingberg, K. Ridell, S. Brogårdh-Roth, M. Vall, H. Berlin

**Affiliations:** 1 Department of Pediatric Dentistry, Faculty of Odontology, Malmö University, SE 205 06 Malmö, Sweden; 2 Library Services, Malmö University, Malmö, Sweden

**Keywords:** Local anaesthesia, Local analgesia, Dental, Systematic review, Child, Adolescent

## Abstract

**Purpose:**

To evaluate the evidence supporting effects and adverse effects of local analgesia using different pharmacological agents and injection techniques during dental treatment in children and adolescents aged 3–19 years.

**Methods:**

A systematic literature search of databases including PubMed, Cochrane, and Scopus was conducted in November 2016. The PRISMA-statement was followed. Two review authors independently assessed the selected randomised control trials for risk of bias and quality.

**Results:**

725 scientific papers were identified. 89 papers were identified to be read in full text of which 80 were excluded. Finally, 9 papers were evaluated for quality and risk of bias. Many of the included papers had methodological shortcomings affecting the possibility to draw conclusions. Information about ethical clearance and consent were missing in some of the included papers. No alarming adverse effects were identified. One study was assessed as having low risk of bias. This reported inferior alveolar nerve block to be more effective than buccal infiltration for dental treatment of mandibular molars, while no differences were found regarding pharmacological agents.

**Conclusions:**

At present, there is insufficient evidence in support of any pharmacologic agent or injection technique as being superior compared to others. There is a need for more rigorous studies which also handle the ethical issues of including children in potentially painful studies.

**Electronic supplementary material:**

The online version of this article (doi:10.1007/s40368-017-0302-z) contains supplementary material, which is available to authorised users.

## Introduction

Pain is defined by IASP (The International Association for the Study of Pain) as “an unpleasant sensory and emotional experience associated with actual or potential tissue damage, or described in terms of such damage” (IASP). According to the same definition, pain is always subjective. There is a risk of pain in conjunction with dental treatment especially when local analgesia is not used or when it does not give the expected full effect. Pain during treatment is problematic as it increases the risk of development of dental anxiety (Klingberg and Broberg 2007) and unfortunately, there are studies where even child patients report painful experiences at the dentists (e.g. Raadal et al. 2002). Thus, it is essential to prevent and reduce the risk of pain whenever possible. However, there is limited knowledge about how efficient different pharmacological agents and injection techniques are to minimise or prevent pain during common dental treatment in child patients.

The purpose of this literature review was to evaluate the evidence supporting effects and adverse effects of local analgesia using different pharmacological agents and injection techniques in children and adolescents aged 3–19 years during dental treatment.

## Materials and methods

### Inclusion criteria

The following research questions were addressed:

- What is the most effective dental local analgesic (agents: lidocaine, mepivacaine, prilocaine, bupivacaine, articaine) for dental treatment (filling therapy, pulp therapy, extractions) in children and adolescents aged 3–19 years?
- What is the most effective injection technique (infiltration, block injection, intraligamentary, intra-osseous) for reducing pain during dental treatment (filling therapy, pulp therapy, extractions) in children and adolescents aged 3–19 years?
- What side-effects and adverse effects occur when administering dental local analgesia for dental treatment in children and adolescents aged 3–19 years?

A PICO model (participants, interventions, control, outcome) was constructed:

Participants

- Children and adolescents, aged 3–19 years

Interventions

- Dental treatment under local analgesia
  - Different pharmacological agents
  - Different injection techniques

Control

- Dental treatment under local analgesia
  - Different pharmacological agents
  - Different injection techniques

Outcome measures

- Pain during dental treatment assessed by the child patient during or directly after treatment using Visual Analogue Scale (VAS), Facial Pain Scale, Wong-Baker FACES^®^ Pain Rating Scale, Eland Colour Scale, or other facial scales
- Adverse effects, side-effects

Type of studies

- Randomised control trials (RCT), systematic reviews (not narrative)

### Exclusion criteria

- Participants 20 years or older, where data could not be extracted for 3–19 year-olds
- Disability or medical conditions
- Surgical interventions other than tooth extractions
- Other local analgesia techniques than infiltration, block injection, intraligamentary, intra-osseous
- Treatment under sedation or general analgesia
- Pain assessment by proxy
- Pain assessment only related to injection
- Other languages than English, Swedish, Danish or Norwegian

### Literature search strategy

**Fig. 1:**
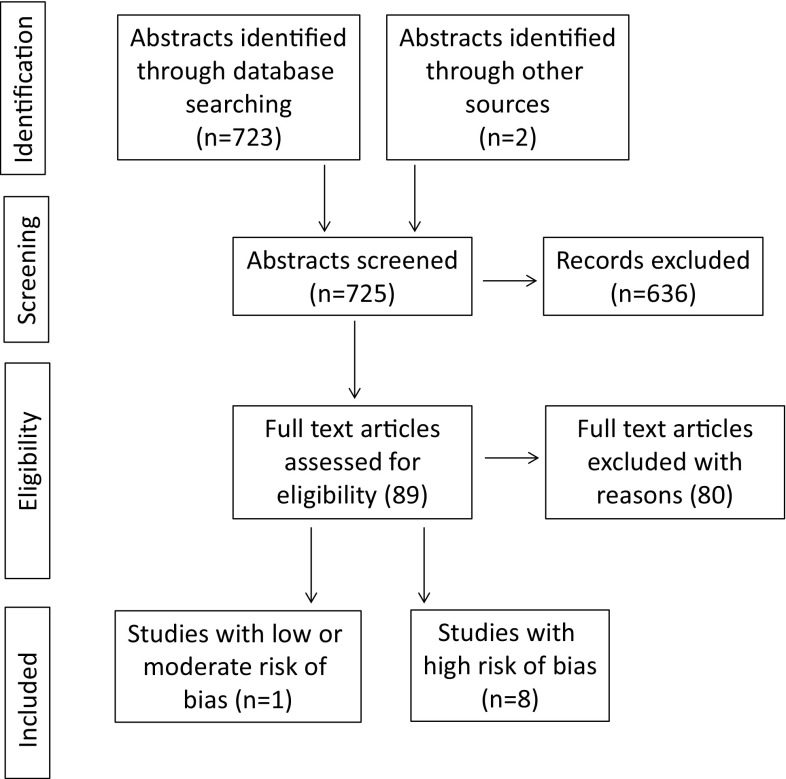
Flow diagram showing the literature review process

To identify studies a systematic search of the literature was conducted using PubMed via NML (29th November 2016), Cochrane via Wiley Online Library (29th November 2016) and Scopus via Elsevier (29th November 2016) (search strategies are presented in supplemental file S1). Limitations were set to randomised control studies, publications in English and publication year 1990 or later. The search was made together with a librarian (MV) specialised in informatics at the library of Malmö University. A total of 471 papers were identified via PubMed, 690 via Cochrane, and 615 via Scopus. After removing duplicates, 723 articles were finally evaluated according to the framework of the PRISMA-statement (Moher et al. 2009). The number of abstracts retrieved, included and excluded articles and the stage of exclusion are shown in a flow-chart (Fig. 1).

All abstracts were screened independently by two review authors (either GK, SBR; or KR, HB) according to the defined inclusion and exclusion criteria. If at least one reviewer considered an abstract relevant, the paper was included and its full text was read.

### Data extraction and quality assessment

The review authors used the same pairs (GK, SBR; and KR, HB) for assessment of relevance of all full text papers. Assessments were made independently and any differences were settled by consensus discussion. Excluded full text papers are shown in supplemental file S2. Quality, as well as data extraction from included studies were assessed independently by all four authors (GK, SBR, KR, HB); again, any differences were resolved by consensus discussion. A set protocol for assessment of randomised studies available from the Swedish Agency for Health Technology Assessment and Assessment of Social Services (SBU) was used to evaluate risk of bias. Low risk of bias was defined as reaching low risk of bias in at least four of the six assessed domains (selection bias, performance bias, detection bias, attrition bias, reporting bias and conflict of interest). For medium risk of bias at least three domains should reach low risk of bias.

A hand-search of the reference lists of all included papers as well as of recently published journals in paediatric dentistry was conducted in February 2017. Two additional relevant publications of interest were found resulting in a total of 725 included papers. The two new articles were assessed using the same criteria.

## Results

### Literature search

Nine studies met the inclusion criteria and risk of bias were assessed. There were four studies comparing different pharmacological agents (Wilson et al. 1990; Malamed et al. 2000a, b; Thakare et al. 2014). Two of these (Malamed et al. 2000a, b) reported data from the same study and study population and were therefore assessed together. Two studies (Oztas et al. 2005; Elbay et al. 2016) compared injection techniques. One study (Arrow 2012) presented results separately for both injection technique and pharmacological agents and was assessed independently for the two interventions, technique and agent, respectively. Finally, there were two studies (Arali and Prasanna 2015; Chopra et al. 2016) that evaluated both technique and agents but without presenting data separately. All studies but one (Oztas et al. 2005) described ethical clearance. Three studies reported sample size calculations (Arrow 2012; Chopra et al. 2016; Elbay et al. 2016).

Articaine was compared with lidocaine in five studies (Malamed et al. 2000a, b; Arrow 2012; Arali and Prasanna 2015; Chopra et al. 2016). Thakare et al. (2014) compared articaine and bupivacaine while Wilson et al. (1990) compared 1 and 2% solutions of lidocaine. The studies on injection techniques used articaine (Elbay et al. 2016) and lidocaine (Oztas et al. 2005). The following injection techniques were studied: inferior alveolar nerve block (IANB); buccal infiltration (BI); intraligamentary/periodontal ligament technique; and supraperiosteal technique.

The study by Arrow (2012) was considered as having low risk of bias, whereas the remaining eight studies were all assessed as having high risk of bias (Table 1). The assessments of risk of bias in the different dimensions are presented in Table 2. Most problems were related to reporting bias where only one study (Arrow 2012) had a published study protocol, and to performance bias.

**Table 1:**
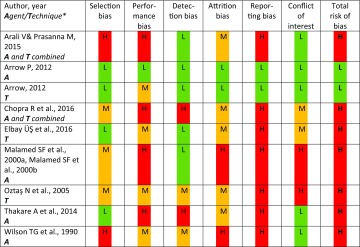
Characteristics and quality assessment of the included studies

*L* low risk of bias in green, *M* medium risk of bias in yellow, *H* high risk of bias in red

* main focus of the included studies LA agent (A) or injection technique (T)

**Table 2:**
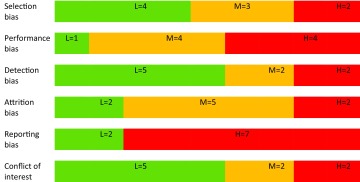
Characteristics and quality assessment of the included studies shown as risk of bias in the six different domains

*L* low risk of bias in green, *M* medium risk of bias in yellow, *H* high risk of bias in red

The included study with low risk of bias (Arrow 2012) was terminated earlier than planned based on a planned interim analysis after gathering data of 50 patients. This decision was made by the study’s Data and Safety Monitoring Committee (DSMC) based on findings showing that some of the treatments were painful and that the success rate (not patient-reported outcome) was higher for IANB than BI, with no difference between the two investigated pharmacological agents. The study was originally designed, based on sample size estimation, to include 350 participants in each arm of the trial. This is a very high number. The other studies included between 25 and 90 children.

### Effect of injection technique or pharmacological agent

Characteristics and results from the study with low risk of bias are shown in Table 3. The study evaluated non-urgent restorative treatment of contralateral teeth in the mandible (lower first or second permanent molars, or second primary molars). Children reported no or mild pain during dental treatment more frequently when having inferior alveolar nerve block (IANB) compared with buccal infiltration (BI) (p = 0.02), while no differences were found when comparing articaine 4% + 1:100 000 adrenaline to lidocaine 2% + 1:80 000 adrenaline.

**Table 3:** Characteristics and quality assessment of the included study with low risk of bias

Author year, country	Study design	Population, patient characteristics	Intervention	Control	Method for evaluation	Risk of bias, comments	Results
Arrow (2012), Australia	RCT Parallel design for LA technique: each participant was administered the analgesic using only one LA technique Split mouth for LA agents: each participant was administered both types of analgesic on separate occasions Study terminated after interim analysis at the accrual of the first 50 patients by DSMC	57 children^a^ Mean age 12.4 years Treated by 6 clinicians from 5 clinics Dental treatment: non-urgent restorative treatment of contralateral teeth in mandible (lower first or second permanent molars, or second primary molars)	IANB: N = 29^b^ Males: 13 Females: 16 Mean age: 11.9 years Articaine 4% + 1:100 000 adrenaline: N = 57 Mean age: 12.4 years Males: 10 Females: 19	BI: N = 28 Males: 8 Females: 20 Mean age: 12.9 years Lidocaine 2% + 1:80 000 adrenaline: N = 57 Mean age: 12.4 years Males: 11 Females: 17	Faces Pain Scale^c^ after treatment	Technique: Low Power calculation Randomisation well described Published study protocol Patient nor clinician blinded for technique	Technique: IANB vs. BI: no/mild pain: 45 vs. 32 Moderate/severe pain: 11 vs. 22 p = 0.02 Chi square test
						LA agent: Low Power calculation Randomisation well described Published study protocol Patient and clinician blinded for LA agent	LA agent: articaine 4% vs. lidocaine 2%: no/mild pain: 40 vs. 37 moderate/ severe pain: 15 vs. 18 p = 0.53 Chi square test

*DSMC* data and safety monitoring committee, *IANB* inferior alveolar block injection, *BI* buccal infiltration

^a^ Originally designed to include 350 children in two arms

^b^ = 1 failed to attend visit 2

^c^ Hicks et al. (2001)

Based on the combination of only one study with low risk of bias and that this study was terminated earlier, it was decided to not formulate evidence-graded results. Still, the quality of evidence should be commented on. As the study was terminated already after an interim analysis of the first included 50 patients because of a large negative effect size, i.e. high frequency of negative outcomes (painful events) it could be argued to add points in GRADE for effect size. On the other hand, the study should probably be found to have too few observations (patients) to conclude that there were no differences in the comparisons (low precision and deduction of points in GRADE) (Balshem et al. 2011; Guyatt et al. 2011).

### Complications and side-effects

Complications and side-effects were evaluated during all stages of data extraction, i.e. also based on abstracts for papers that were not read in full text. There were no serious side-effects or adverse effects reported apart from cases described as soft tissue injuries such as lip or cheek biting, or pain related to injection site or type of dental treatment.

### Ethics

Ethical aspects are essential in the concept of systematic reviews and considerations of ethical aspects were based on Heintz et al. (2015). Even though it is not possible, based on this systematic review, to determine if there are any health benefits from the use of local analgesia apart from pain reduction it should be acknowledged that the relationship between painful events, developing of dental anxiety and deterioration of oral health are well known already since the 1980s (Berggren 1984). The main ethical issues identified in this systematic review concern compatibility with ethical norms, and the autonomy, integrity/privacy of children as patients. It is important that children and adolescents be not excluded from research that can be beneficial for them. At the same time, it could be questioned if there already is sufficient knowledge about local analgesia based on research in adults. This was not investigated in this survey. In all research involving young individuals, specific protections to safeguard children’s rights and welfare are therefore necessary (CIOMS 2016). The combination of the inclusion of very young children and problems related to study design is an ethical issue. There is probably a need for more rigorous studies foremost in adults, but also studies that consider the perspective of the child.

## Discussion

This systematic review was undertaken to evaluate effects and adverse effects of local analgesia using different and injection techniques in children during dental treatment. It has shown that there is an inadequate scientific basis for evaluation of the effect of pharmacological agents as well as injection techniques. Importantly, no reports of serious side-effects or adverse effects were found.

The literature search in three data bases resulted in 725 abstracts of which only nine were found to be relevant and included for assessment of quality. One reason for the low number of included studies was the inclusion criterion “pain during dental treatment assessed by the child patient” that was only met by relatively few studies. This criterion was considered central based on the strong position of the patients’ understanding and perceptions in the definition of pain (IASP). Accordingly, patient-reported and patient-centred outcomes should be used when assessing pain. It may be argued that young children are not able to use such pain rating methods (visual analogue scales, facial scales or colour scales). However, as pain is potentially harmful it must be questioned if pain studies should at all be carried out in individuals too young to able to understand and complete pain measurements.

Children as young as 4-years-old were included in the identified studies. This is a very young age and it must be questioned if it is a good ethical standard to carry out any dental RCT in such young children. Ethical clearance was not described in one study (Oztas et al. 2005) and information about how children were informed about the study was generally lacking except for one study (Wilson et al. 1990). Six studies (Wilson et al. 1990; Arrow 2012; Thakare et al. 2014; Arali and Prasanna 2015; Chopra et al. 2016; Elbay et al. 2016) reported consent procedures with parents or child patients (age appropriate), while the remaining studies did not describe anything about consent. It is possible that these ethical problems were all cleared in the clinical trial situations, but for some reason not described in the articles. This is extremely problematic and sends a strong signal to all involved in the process of reviewing and editing scientific papers on dental research in children and adolescents. This information must be specified in the manuscripts. It is advocated that studies evaluating the effect of local analgesia (different agents or injection techniques) target primarily adult patients competent to consent participation. Based on outcomes from such studies also younger populations like adolescents and older children may be studied provided there is a relevant research question that cannot be answered unless young individuals are included, that there is a solid study design and careful assent and consent procedures (CIOMS 2016).

The quality of the included studies was unfortunately not of a high standard. There were general problems concerning sample size calculations, descriptions of randomisation, ensuring equal dental treatment in both study arms, blinding, and publication of study protocols. The latter should be a prerequisite for publication of any RCT today. These aspects of study quality are even more important when including children in clinical studies, and essential when pain is at stake. The only identified high quality study (Arrow 2012) was terminated earlier than planned. This tough but significant decision was in fact enabled because of an existing published study protocol. It is thereby a good example of the importance of well-designed studies with published and available protocols.

Arrow (2012) reported better results, less pain, when using IANB than BI for restorative treatment in the lower jaw (first or second permanent molars, or second primary molars). As a precautionary principle, the findings of more pain when using BI regardless of what pharmacological agent should be kept in mind. The study was considered a low risk of bias study but quality assessment using GRADE was not done. Thus, it is not possible to formulate any evidence based on these results.

It is not possible to use results from this systematic review for guidelines. However, based on the results, empiricism and clinical experience a few statements can be made:

- Pain in conjunction with dental treatment in children and adolescents should be avoided and minimised
- Local analgesia with available pharmacological agents (lidocaine, prilocaine, and articaine) are effective local analgesics for prevention of pain during dental treatment.
- There is no evidence of any injection techniques being more effective than others to reduce pain during dental treatment.

## Conclusions

There are ethical issues of including children in studies on local analgesia. The literature search identified one publication with a low risk of bias. Based on this study, we cannot determine whether any specific pharmacological agent or injection technique is more effective than others for pain management in dental treatment of children and adolescents. Further randomised clinical trials with appropriate sample sizes, study design should be completed provided ethical issues are managed.

## Electronic supplementary material

Below is the link to the electronic supplementary material.

Supplementary material 1 (DOCX 27 kb)

Supplementary material 2 (DOCX 28 kb)
