# Supplementary material for: Local analgesia in paediatric dentistry: a systematic review of techniques and pharmacologic agents
Source: Eur Arch Paediatr Dent. 2017 Sep 14;18(5):323–9. doi: 10.1007/s40368-017-0302-z (PMC5651714; doi:10.1007/s40368-017-0302-z)
Supplement: Supplementary file 1 — Supplementary material 1 (DOCX 27 kb) [file 40368_2017_302_MOESM1_ESM.docx]

**S1. Search strategies**

**PubMed via NML, 29th November 2016:**

|  | **Search term** | **Items found** |
| --- | --- | --- |
| Population | Children and adolesents 3-19 yrs |  |
|  | “Child”[MeSH] | 1686924 |
|  | “Adolescent”[MeSH] | 1759044 |
|  | “Minors”[MeSH] | *2364* |
|  | Child[tiab] | *327425* |
|  | Children[tiab] | 860844 |
|  | Adolescent[tiab] | 94681 |
|  | Adolescence[tiab] | 57616 |
|  | Minors[tiab] | 3021 |
|  | Teenager[tiab] | 2158 |
|  | Teenagers[tiab] | 10476 |
|  | Young[tiab] | 388719 |
|  | Offspring[tiab] | 56505 |
|  | Juvenile[tiab] | 62736 |
|  | Puberty[tiab] | 24387 |
|  | School age[tiab] | 10869 |
|  | Boys[tiab] | 72125 |
|  | Girls[tiab] | 76693 |
|  | 1 OR 2 OR 3 OR 4 OR 5 OR 6 OR 7 OR 8 OR 9 OR 10 OR 11 OR 12 OR 13 OR 14 OR 15 OR 16 OR 17 | **3258255** |
| Intervention | Local anesthesia |  |
|  | “Anesthetics, Local”[MeSH] | 30312 |
|  | “Anesthesia, Dental”[MeSH] | 10498 |
|  | “Anesthesia, Local”[MeSH] | 15772 |
|  | “Carticaine”[MeSH] | 467 |
|  | “Mepivacaine”[MeSH] | 1912 |
|  | “Lidocaine”[MeSH] | 22629 |
|  | “Bupivacaine”[MeSH] | 10789 |
|  | “Prilocaine”[MeSH] | 2032 |
|  | Anesthesia, Local[tiab] | 33754 |
|  | Anaesthesia, Local[tiab] | 33754 |
|  | Local Anesthesia[tiab] | 12048 |
|  | Local Anaesthesia[tiab] | 4703 |
|  | Anesthesia, Dental[tiab] | 6750 |
|  | Anaesthesia, Dental[tiab] | 6750 |
|  | Dental Anesthesia[tiab] | 312 |
|  | Dental Anaesthesia[tiab] | 267 |
|  | Anesthesia, Infiltration[tiab] | 3379 |
|  | Anaesthesia, Infiltration[tiab] | 3379 |
|  | Infiltration Anesthesia[tiab] | 398 |
|  | Infiltration, Anaesthesia[tiab] | 155 |
|  | Anesthetic, Local[tiab] | 28018 |
|  | Anaesthetic, Local[tiab] | 1 |
|  | Local Anesthetic[tiab] | 10246 |
|  | Local Anaesthetic | 5081 |
|  | Articain*[tiab] | 424 |
|  | Bupivacain*[tiab] | 11415 |
|  | Carbocain*[tiab] | 121 |
|  | Carbostesin*[tiab] | 28 |
|  | Carticain*[tiab] | 59 |
|  | Citanest[tiab] | 124 |
|  | Lidocain*[tiab] | 19603 |
|  | Lignocain*[tiab] | 2689 |
|  | Mepivacain*[tiab] | 1612 |
|  | Marcain*[tiab] | 359 |
|  | Prilocain*[tiab] | 1440 |
|  | Propitocain*[tiab] | 16 |
|  | Sensorcain*[tiab] | 12 |
|  | Septocain*[tiab] | 4 |
|  | Vadocain*[tiab] | 16 |
|  | Xylocain*[tiab] | 1102 |
|  | Xylonest[tiab] | 19 |
|  | 19 OR 20 OR 21 OR 22 OR 23 OR 24 OR 25 OR 26 OR 27 OR 28 OR 29 OR 30 OR 31 OR 32 OR 33 OR 34 OR 35 OR 36 OR 37 OR 38 OR 39 OR 40 OR 41 OR 42 OR 43 OR 44 OR 45 OR 46 OR 47 OR 48 OR 49 OR 50 OR 51 OR 52 OR 53 OR 54 OR 56 OR 57 OR 58 OR 59 | **100954** |
| Intervention | Dental treatment |  |
|  | “Root Canal Therapy” [MeSH] | 18743 |
|  | “Dental Cavity Preparation”[MeSH] | 7411 |
|  | “Endodontics”[MeSH] | 25090 |
|  | “Pulpotomy”[MeSH] | 1369 |
|  | “Pulpectomy”[MeSH] | 1086 |
|  | “Dental Restoration, Permanent”[MeSH] | 22185 |
|  | “Dental Pulp Capping” [MeSH] | 1871 |
|  | “Tooth Extraction”[MeSH] | 18115 |
|  | “Dental Care”[MeSH] | 29864 |
|  | Dental Care[tiab] | 10325 |
|  | Dental Treatment[tiab] | 6641 |
|  | Teeth Extraction*[tiab] | 1087 |
|  | Tooth Extraction*[tiab] | 3967 |
|  | Pulp Capping[tiab] | 973 |
|  | Pulp Therapy[tiab] | 402 |
|  | Filling Therapy[tiab] | 60 |
|  | Dental Restoration[tiab] | 593 |
|  | Pulpectomy[tiab] | 398 |
|  | Pulpotomy[tiab] | 854 |
|  | Endodontic*[tiab] | 16044 |
|  | Cavity Prep*[tiab] | 1910 |
|  | Root Canal[tiab] | 12572 |
|  | 61 OR 62 OR 63 OR 64 OR 65 OR 66 OR 67 OR 68 OR 69 OR 70 OR 71 OR 72 OR 73 OR 74 OR 75 OR 76 OR 77 OR 78 OR 79 OR 80 OR 81 OR 82 | **113841** |
|  | **18 AND 60 AND 83** | **2477** |
| Study design | Cochrane sensitivity- and precision-maximizing version (2008 revision) PubMed format. |  |
|  | (randomized controlled trial[pt] OR controlled clinical trial[pt] OR randomized[tiab] OR placebo[tiab] OR clinical trials as topic[mesh:noexp] OR randomly[tiab] OR trial[ti] NOT (animals[mh] NOT humans [mh])) | 986414 |
|  | **18 AND 60 AND 83 AND 85** | 554 |
| Limits |  |  |
|  | Publication date from 1990/01/01; English, Norweigan, Danish, Swedish |  |
|  | **18 AND 60 AND 83 AND 85 AND 87** | **471** |

**Cochrane via Wiley Online Library, 29th November 2016:**

|  | **Search term** | **Items found** |
| --- | --- | --- |
| Population | Children and adolescents 3-19 yrs |  |
|  | MeSH descriptor: [Child] explode all trees | 208 |
|  | MeSH descriptor: [Adolescent] explode all trees | 87541 |
|  | MeSH descriptor: [Minors] explode all trees | 9 |
|  | child:ti,ab,kw (Word variations have been searched) | 90928 |
|  | children:ti,ab,kw (Word variations have been searched) | 90928 |
|  | adolescent:ti,ab,kw (Word variations have been searched) | 107266 |
|  | adolescence:ti,ab,kw (Word variations have been searched) | 3187 |
|  | minors:ti,ab,kw (Word variations have been searched) | 10734 |
|  | teenager:ti,ab,kw (Word variations have been searched) | 455 |
|  | teenagers:ti,ab,kw (Word variations have been searched) | 455 |
|  | young:ti,ab,kw (Word variations have been searched) | 71231 |
|  | offspring:ti,ab,kw (Word variations have been searched) | 714 |
|  | juvenile:ti,ab,kw (Word variations have been searched) | 1725 |
|  | puberty:ti,ab,kw (Word variations have been searched) | 736 |
|  | school age:ti,ab,kw (Word variations have been searched) | 8732 |
|  | boys:ti,ab,kw (Word variations have been searched) | 4212 |
|  | girls:ti,ab,kw (Word variations have been searched) | 4188 |
|  | #1 or #2 or #3 or #4 or #5 or #6 or #7 or #8 or #9 or #10 or #11 or #12 or #13 or #14 or #15 or #16 or #17 | **212198** |
| Intervention | Local anesthesia |  |
|  | MeSH descriptor: [Anesthetics, Local] explode all trees | 6755 |
|  | MeSH descriptor: [Anesthesia, Dental] explode all trees | 978 |
|  | MeSH descriptor: [Anesthesia, Local] explode all trees | 1921 |
|  | MeSH descriptor: [Carticaine] explode all trees | 153 |
|  | MeSH descriptor: [Mepivacaine] explode all trees | 378 |
|  | MeSH descriptor: [Lidocaine] explode all trees | 4116 |
|  | MeSH descriptor: [Bupivacaine] explode all trees | 3745 |
|  | MeSH descriptor: [Prilocaine] explode all trees | 633 |
|  | Anesthesia, local:ti,ab,kw (Word variations have been searched) | 9381 |
|  | Anaesthesia, local:ti,ab,kw (Word variations have been searched) | 9381 |
|  | local anesthesia:ti,ab,kw (Word variations have been searched) | 9381 |
|  | local anaesthesia:ti,ab,kw (Word variations have been searched) | 9381 |
|  | anesthesia, dental:ti,ab,kw (Word variations have been searched) | 1647 |
|  | anaesthesia, dental:ti,ab,kw (Word variations have been searched) | 1647 |
|  | dental anesthesia:ti,ab,kw (Word variations have been searched) | 1647 |
|  | dental anaesthesia:ti,ab,kw (Word variations have been searched) | 1647 |
|  | anesthesia, infiltration:ti,ab,kw (Word variations have been searched) | 1234 |
|  | anaesthesia, infiltration:ti,ab,kw (Word variations have been searched) | 1234 |
|  | infiltration anestesia:ti,ab,kw (Word variations have been searched) | 1234 |
|  | infiltration anaestesia:ti,ab,kw (Word variations have been searched) | 1234 |
|  | anesthetics, local:ti,ab,kw (Word variations have been searched) | 9830 |
|  | anaesthetics, local:ti,ab,kw (Word variations have been searched) | 2490 |
|  | local anesthetics:ti,ab,kw (Word variations have been searched) | 9830 |
|  | local anaesthetics:ti,ab,kw (Word variations have been searched) | 2490 |
|  | articain*:ti,ab,kw (Word variations have been searched) | 212 |
|  | bupivacain*:ti,ab,kw (Word variations have been searched) | 8230 |
|  | carbocain*:ti,ab,kw (Word variations have been searched) | 26 |
|  | carbostesin:ti,ab,kw (Word variations have been searched) | 6 |
|  | carticain*:ti,ab,kw (Word variations have been searched) | 171 |
|  | citanest:ti,ab,kw (Word variations have been searched) | 27 |
|  | lidocain*:ti,ab,kw (Word variations have been searched) | 7963 |
|  | lignocain*:ti,ab,kw (Word variations have been searched) | 1126 |
|  | mepivacain*:ti,ab,kw (Word variations have been searched) | 762 |
|  | marcain*:ti,ab,kw (Word variations have been searched) | 126 |
|  | prilocain*:ti,ab,kw (Word variations have been searched) | 964 |
|  | propitocain*:ti,ab,kw (Word variations have been searched) | 1 |
|  | septocain*:ti,ab,kw (Word variations have been searched) | 1 |
|  | sensorcain*:ti,ab,kw (Word variations have been searched) | 8 |
|  | vadocain*:ti,ab,kw (Word variations have been searched) | 5 |
|  | xylocain*:ti,ab,kw (Word variations have been searched) | 203 |
|  | xylonest:ti,ab,kw (Word variations have been searched) | 6 |
|  | #19 or #20 or #21 or #22 or #23 or #24 or #25 or #26 or #27 or #28 or #29 or #30 or #31 or #32 or #33 or #34 or #35 or #36 or #37 or #38 or #39 or #40 or #41 or #42 or #43 or #44 or #45 or #46 or #47 or #48 or #49 or #50 or #51 or #52 or #53 or #54 or #55 or #56 or #57 or #58 or #59 | **21867** |
| Intervention | Dental tretament |  |
|  | MeSH descriptor: [Root Canal Therapy] explode all trees | 978 |
|  | MeSH descriptor: [Dental Cavity Preparation] explode all trees | 617 |
|  | MeSH descriptor: [Endodontics] explode all trees | 1213 |
|  | MeSH descriptor: [Pulpotomy] explode all trees | 114 |
|  | MeSH descriptor: [Pulpectomy] explode all trees | 66 |
|  | MeSH descriptor: [Dental Restoration, Permanent] explode all trees | 1372 |
|  | MeSH descriptor: [Dental Pulp Capping] explode all trees | 91 |
|  | MeSH descriptor: [Tooth Extraction] explode all trees | 1489 |
|  | MeSH descriptor: [Dental Care] explode all trees | 565 |
|  | dental care:ti,ab,kw (Word variations have been searched) | 2314 |
|  | dental treatment:ti,ab,kw (Word variations have been searched) | 7879 |
|  | tooth extraction:ti,ab,kw (Word variations have been searched) | 2387 |
|  | pulp capping:ti,ab,kw (Word variations have been searched) | 169 |
|  | pulp therapy:ti,ab,kw (Word variations have been searched) | 377 |
|  | filling therapy:ti,ab,kw (Word variations have been searched) | 1795 |
|  | dental restoration:ti,ab,kw (Word variations have been searched) | 2945 |
|  | pulpectomy:ti,ab,kw (Word variations have been searched) | 132 |
|  | pulpotomy:ti,ab,kw (Word variations have been searched) | 178 |
|  | endodontic*:ti,ab,kw (Word variations have been searched) | 939 |
|  | cavity preparation:ti,ab,kw (Word variations have been searched) | 1380 |
|  | root canal:ti,ab,kw (Word variations have been searched) | 1557 |
|  | #59 or #60 or #61 or #62 or #63 or #64 or #65 or #66 or #67 or #68 or #69 or #70 or #71 or #72 or #73 or #74 or #75 or #76 or #77 or #78 or #79 or #80 or #81 | **15236** |
|  | **18 AND 60 AND 82** | **690** |
| Study design |  |  |
|  | Trials | **683** |
|  | Cochrane Reviews | 7 |
| Limits |  |  |
|  | Publication date from 1990/01/01; | **690** |

**Scopus via Elsevier, 29th November 2016**

|  | **Search term** | **Items found** |
| --- | --- | --- |
| Population | Children and adolesents 3-19 yrs |  |
|  | TITLE-ABS-KEY ( child ) | 2,673,495 |
|  | TITLE-ABS-KEY ( children ) | 2,673,526 |
|  | TITLE-ABS-KEY ( adolescent ) | 2,035,187 |
|  | TITLE-ABS-KEY ( adolescence ) | 105,020 |
|  | TITLE-ABS-KEY ( minors ) | 393,289 |
|  | TITLE-ABS-KEY ( teenager ) | 21,101 |
|  | TITLE-ABS-KEY ( teenagers ) | 21,101 |
|  | TITLE-ABS-KEY ( young ) | 1,405,741 |
|  | TITLE-ABS-KEY ( puberty ) | 46,201 |
|  | TITLE-ABS-KEY ( offsprig ) | 88,895 |
|  | TITLE-ABS-KEY ( juvenile ) | 177,139 |
|  | TITLE-ABS-KEY ( ”school age” ) | 16,457 |
|  | TITLE-ABS-KEY ( boys ) | 173,386 |
|  | TITLE-ABS-KEY ( girls ) | 179,992 |
|  | #1 OR #2 OR #3 OR #4 OR #5 OR #6 OR #7 OR #8 OR #9 OR #10 OR #11 OR #12 OR #13 OR #14 | **5,126,920** |
|  |  |  |
| Intervention | Dental treatment |  |
|  | TITLE-ABS-KEY ( ”Dental Care” ) | 68,158 |
|  | TITLE-ABS-KEY ( ”Dental Treatment” ) | 8,504 |
|  | TITLE-ABS-KEY ( ”Tooth extraction” ) | 23,827 |
|  | TITLE-ABS-KEY ( ”Teeth extraction” ) | 23,827 |
|  | TITLE-ABS-KEY ( ”Pulp Capping” ) | 2,375 |
|  | TITLE-ABS-KEY ( ”Pulp Therapy” ) | 481 |
|  | TITLE-ABS-KEY ( ”Filling Therapy” ) | 68 |
|  | TITLE-ABS-KEY ( ”Dental Restoration” ) | 27,648 |
|  | TITLE-ABS-KEY ( Pulpectomy ) | 1,636 |
|  | TITLE-ABS-KEY ( Pulpotomy ) | 1,636 |
|  | TITLE-ABS-KEY ( endodontic* ) | 37,293 |
|  | TITLE-ABS-KEY ( ”Cavity preparation” ) | 8,380 |
|  | TITLE-ABS-KEY ( ”Root Canal” ) | 28,835 |
|  | #16 OR #17 OR #18 OR #19 OR #20 OR #21 OR #22 OR #23 OR #24 OR #25 OR #26 OR #27 OR #28 | 188,524 |
| Intervention | Local anesthesia |  |
|  | TITLE-ABS-KEY ( ”Local anesthesia” ) | 39,897 |
|  | TITLE-ABS-KEY ( ”Local anaesthesia” ) | 39,897 |
|  | TITLE-ABS-KEY ( ”Dental anesthesia” ) | 10,055 |
|  | TITLE-ABS-KEY ( ”Dental anaesthesia” ) | 10,055 |
|  | TITLE-ABS-KEY ( ”Infiltration anesthesia” ) | 787 |
|  | TITLE-ABS-KEY ( ”Infiltration anaesthesia” ) | 787 |
|  | TITLE-ABS-KEY ( ”Local Anesthetic” ) | 39,960 |
|  | TITLE-ABS-KEY ( ” Local Anaesthetic” ) | 9,381 |
|  | TITLE-ABS-KEY ( Articain* ) | 996 |
|  | TITLE-ABS-KEY (Bupivacain* ) | 32,086 |
|  | TITLE-ABS-KEY ( Carbocain* ) | 606 |
|  | TITLE-ABS-KEY ( Carbostesin ) | 259 |
|  | TITLE-ABS-KEY ( carticain* ) | 507 |
|  | TITLE-ABS-KEY ( citanest ) | 444 |
|  | TITLE-ABS-KEY ( Lidocain* ) | 68,740 |
|  | TITLE-ABS-KEY ( Lignocain* ) | 3,476 |
|  | TITLE-ABS-KEY ( Marcain* ) | 2,188 |
|  | TITLE-ABS-KEY ( Mepivacain* ) | 6,138 |
|  | TITLE-ABS-KEY ( Prilocain* ) | 4,862 |
|  | TITLE-ABS-KEY ( Propitocain* ) | 39 |
|  | TITLE-ABS-KEY ( Sensorcain*) | 135 |
|  | TITLE-ABS-KEY ( Septocain* ) | 33 |
|  | TITLE-ABS-KEY ( Vadocain* ) | 25 |
|  | TITLE-ABS-KEY ( Xylocain* ) | 4,989 |
|  | TITLE-ABS-KEY ( Xylonest ) | 125 |
|  | #30 OR #31 OR #32 OR #33 OR #34 OR #35 OR #36 OR #37 OR #38 OR #39 OR #40 OR #41 OR #42 OR #43 OR #44 OR #45 OR #46 OR #47 OR #48 OR ¤9 OR #50 OR #51 OR #52 OR #53 OR #54 | **150,032** |
|  | #15 AND #29 AND #55 | 2,581 |
| Study design | Filter for RCT |  |
|  | ( INDEXTERMS ( "clinical trials" OR "clinical trials as a topic" OR "randomized controlled trial" OR "Randomized Controlled Trials as Topic" OR "controlled clinical trial" OR "Controlled Clinical Trials" OR "random allocation" OR "Double-Blind Method" OR "Single-Blind Method" OR "Cross-Over Studies" OR "Placebos" OR "multicenter study" OR "double blind procedure" OR "single blind procedure" OR "crossover procedure" OR "clinical trial" OR "controlled study" OR "randomization" OR "placebo" ) ) OR ( TITLE-ABS-KEY ( ( "clinical trials" OR "clinical trials as a topic" OR "randomized controlled trial" OR "Randomized Controlled Trials as Topic" OR "controlled clinical trial" OR "Controlled Clinical Trials as Topic" OR "random allocation" OR "randomly allocated" OR "allocated randomly" OR "Double-Blind Method" OR "Single-Blind Method" OR "Cross-Over Studies" OR "Placebos" OR "cross-over trial" OR "single blind" OR "double blind" OR "factorial design" OR "factorial trial" ) ) ) OR ( TITLE-ABS ( clinical trial* OR trial* OR rct* OR random* OR blind* ) ) | 6,018,631 |
|  | #56 AND #57 | 739 |
| Limits |  |  |
|  | English, year of publication 1990-, Type of document (articles, reviews) | 615 |
