# Supplementary material for: Local analgesia in paediatric dentistry: a systematic review of techniques and pharmacologic agents
Source: Eur Arch Paediatr Dent. 2017 Sep 14;18(5):323–9. doi: 10.1007/s40368-017-0302-z (PMC5651714; doi:10.1007/s40368-017-0302-z)
Supplement: Supplementary file 2 — Supplementary material 2 (DOCX 28 kb) [file 40368_2017_302_MOESM2_ESM.docx]

**S2. Excluded studies**

**List of excluded full text papers**

| **Reference**  **number** | **Name of first author, year** | **Reason for exclusion** |
| --- | --- | --- |
| 1 | Alamoudi, 2016 | not patient-reported outcome |
| 2 | Al-Kahtani, 2014 | no data for 3-19 yrs |
| 3 | Allen, 2002 | pain assessed during injection |
| 4 | Araujo, 2015 | no data for 3-19 yrs |
| 5 | Asarch, 1999 | pain assessed during injection |
| 6 | Ashraf, 2013 | adult patients |
| 7 | Badcock, 2007 | adult patients |
| 8 | Bahgdadi, 1999 | not dental treatment |
| 9 | Baghlaf, 2015 | pain assessed during injection |
| 10 | Batista, 2010 | no data for 3-19 yrs |
| 11 | Bhananker, 2008 | patient sedated |
| 12 | Biocanin 2013 | adult patients |
| 13 | Ching, 2014 | pain assessed during injection |
| 14 | Cho, 1998 | not dental treatment |
| 15 | Daubländer, 2012 | no data for 3-19 yrs |
| 16 | de Menezes Abreu, 2011 | not evaluating LA |
| 17 | Donkor, 1990 | no data for 3-19 yrs |
| 18 | Donohue, 1993 | patient sedated |
| 19 | Elbay, 2016 | not patient-reported outcome |
| 20 | Fagade, 2005 | no data for 3-19 yrs |
| 21 | Fan, 2009 a | no data for 3-19 yrs |
| 22 | Fan, 2009 b | no data for 3-19 yrs |
| 23 | Forloine, 2010 | no data for 3-19 yrs |
| 24 | Gibson, 2000 | pain assessed during injection |
| 25 | Goodell, 2000 | no data for 3-19 yrs |
| 26 | Gregorio, 2008 | no data for 3-19 yrs |
| 27 | Guglielmo, 2011 | no data for 3-19 yrs |
| 28 | Haghgoo, 2015 | not patient-reported outcome |
| 29 | Hoef, 2007 | not evaluating LA |
| 30 | Hung, 2006 | no data for 3-19 yrs |
| 31 | Isiordia-Espinoza, 2012 | no data for 3-19 yrs |
| 32 | Janjua, 2012 | no data for 3-19 yrs |
| 33 | Jing, 2014 | no data for 3-19 yrs |
| 34 | Joyce, 1993 | no data for 3-19 yrs |
| 35 | Kämmerer, 2012 | adult patients |
| 36 | Kämmerer, 2013 | Not RCT, some patients sedated |
| 37 | Kanaa, 2012 | no data for 3-19 yrs |
| 38 | Kandiah, 2012 | pain assessed during injection |
| 39 | Klein, 2005 | not patient-reported outcome |
| 40 | Kohler, 2008 | no data for 3-19 yrs |
| 41 | Kreimer, 2012 | no data for 3-19 yrs |
| 42 | Krzemiński, 2011 | adult patients |
| 43 | Kuscu, 2008 | pain assessed during injection |
| 44 | Lima, 2013 | no data for 3-19 yrs |
| 45 | Malamed, 2013 | adult patients |
| 46 | Maniglia-Ferreira, 2009 | adult patients |
| 47 | Martínez-Rodríguez, 2012 | surgical treatment |
| 48 | Martinez Gonzalez, 2003 | adult patients |
| 49 | Meechan, 2001 | not evaluation effect of LA during dental treatment |
| 50 | Mellor, 2005 | no data for 3-19 yrs |
| 51 | Mittal, 2015 | pain assessed during injection |
| 52 | Odabas, 2012 | not patient-reported outcome |
| 53 | Oulis, 1996 | not patient-reported outcome |
| 54 | Palm, 2014 | pain assessed during injection |
| 55 | Parirokh, 2010 | no data for 3-19 yrs |
| 56 | Pereira, 2013 | adult patients |
| 57 | Poorni, 2011 | no data for 3-19 yrs |
| 58 | Ram, 2002 | pain assessed during injection |
| 59 | Ram, 2006 a | pain assessed during injection |
| 60 | Ram, 2006 b | patient sedated |
| 61 | Ran, 2003 | patient sedated |
| 62 | Reitz, 1998 a | no data for 3-19 yrs |
| 63 | Reitz, 1998 b | no data for 3-19 yrs |
| 64 | Replogle, 1997 | no data for 3-19 yrs |
| 65 | Rogers, 2014 | no data for 3-19 yrs |
| 66 | Said, 2015 | adult patients |
| 67 | Salomon, 2012 | not RCT |
| 68 | Sampaio, 2012 | adult patients |
| 69 | Sandeep, 2016 | pain assessed during injection |
| 70 | Satish, 2013 | no data for 3-19 yrs |
| 71 | Sharaf, 1997 | not patient-reported outcome |
| 72 | Srinivasan, 2009 | no data for 3-19 yrs |
| 73 | Srisurang, 2011 | no data for 3-19 yrs |
| 74 | Thongkukiatkun, 2015 | no data for 3-19 yrs; not RCT |
| 75 | Tudeshchoie, 2013 | not patient-reported outcome |
| 76 | Uckan, 2006 | no data for 3-19 yrs |
| 77 | Willett, 2008 | adult patients |
| 78 | Yassen, 2010 | not patient-reported outcome |
| 79 | Yilmaz, 2011 | not patient-reported outcome |
| 80 | Zarei, 2012 | adult patients |

**Excluded full text papers, full references**

1. Alamoudi NM, Baghlaf KK, Elashiry EA, Farsi NM, El Derwi DA, Bayoumi AM. The effectiveness of computerized anesthesia in primary mandibular molar pulpotomy: A randomized controlled trial. Quintessence Int. 2016;47:217-24. doi: 10.3290/j.qi.a34977.
2. Al-Kahtani A. Effect of long acting local anesthetic on postoperative pain in teeth with irreversible pulpitis: Randomized clinical trial. Saudi Pharmaceutical Journal. 2014;22:39-42. doi: 10.1016/j.jsps.2013.01.004.
3. Allen KD, Kotil D, Larzelere RE, Hutfless S, Beiraghi S. Comparison of a computerized anesthesia device with a traditional syringe in preschool children. Pediatr Dent. 2002;24:315-20. Accessed 24 November 2016.
4. Araujo GM, Barbalho JC, Dias TG, Santos Tde S, Vasconcellos RJ, de Morais HH. Comparative analysis between computed and conventional inferior alveolar nerve block techniques. J Craniofac Surg. 2015;26:e733-6. doi: 10.1097/SCS.0000000000002245.
5. Asarch T, Allen K, Petersen B, Beiraghi S. Efficacy of a computerized local anesthesia device in pediatric dentistry. Pediatr Dent. 1999;21:421-4.
6. Ashraf H, Kazem M, Dianat O, Noghrehkar F. Efficacy of articaine versus lidocaine in block and infiltration anesthesia administered in teeth with irreversible pulpitis: A prospective, randomized, double-blind study. J Endod. 2013;39:6-10. doi: 10.1016/j.joen.2012.10.012.
7. Badcock ME, Gordon I, McCullough MJ. A blinded randomized controlled trial comparing lignocaine and placebo administration to the palate for removal of maxillary third molars. Int J Oral Maxillofac Surg. 2007;36:1177-82. doi: 10.1016/j.ijom.2007.06.001.
8. Baghdadi ZD. Evaluation of electronic dental anesthesia in children. Oral Surg Oral Med Oral Pathol Oral Radiol Endod. 1999;88:418-23. doi: S1079-2104(99)70055-7.
9. Baghlaf K, Alamoudi N, Elashiry E, Farsi N, El Derwi DA, Abdullah AM. The pain-related behavior and pain perception associated with computerized anesthesia in pulpotomies of mandibular primary molars: A randomized controlled trial. Quintessence Int. 2015;46:799-806. doi: 10.3290/j.qi.a34553.
10. Batista da SC, Berto LA, Volpato MC, et al. Anesthetic efficacy of articaine and lidocaine for incisive/mental nerve block. J Endod. 2010;36:438-41. doi: 10.1016/j.joen.2009.12.014.
11. Bhananker SM, Azavedo LF, Splinter WM. Addition of morphine to local anesthetic infiltration does not improve analgesia after pediatric dental extractions. Paediatr Anaesth. 2008;18:140-4. doi: 10.1111/j.1460-9592.2007.02399.x.
12. Biocanin V, Brkovic B, Milicic B, Stojic D. Efficacy and safety of intraseptal and periodontal ligament anesthesia achieved by computer-controlled articaine + epinephrine delivery: A dose-finding study. Clin Oral Investig. 2013;17:525-33. doi: 10.1007/s00784-012-0724-2.
13. Ching D, Finkelman M, Loo CY. Effect of the DentalVibe injection system on pain during local anesthesia injections in adolescent patients. Pediatr Dent. 2014;36:51-5.
14. Cho SY, Drummond BK, Anderson MH, Williams S. Effectiveness of electronic dental anesthesia for restorative care in children. Pediatr Dent. 1998;20:105-11.
15. Daubländer M, Kämmerer PW, Willershausen B, et al. Clinical use of an epinephrine-reduced (1/400,000) articaine solution in short-time dental routine treatments-a multicenter study. Clin Oral Investig. 2012;16:1289-95. Accessed 24 November 2016. doi: 10.1007/s00784-011-0608-x.
16. de Menezes Abreu DM, Leal SC, Mulder J, Frencken JE. Pain experience after conventional, atraumatic, and ultraconservative restorative treatments in 6- to 7-yr-old children. Eur J Oral Sci. 2011;119:163-8. doi: 10.1111/j.1600-0722.2011.00806.x.
17. Donkor P, Wong J, Punnia-Moorthy A. An evaluation of the closed mouth mandibular block technique. Int J Oral Maxillofac Surg. 1990;19:216-9.
18. Donohue D, Garcia GF, King DL, Barnwell GM. Evaluation of mandibular infiltration versus block anesthesia in pediatric dentistry. ASDC J Dent Child. 1993;60:104-6.
19. Elbay US, Elbay M, Kaya E, Yildirim S. Effects of two different anesthetic solutions on injection pain, efficacy, and duration of soft-tissue anesthesia with inferior alveolar nerve block for primary molars. J Clin Pediatr Dent. 2016;40:456-63. doi: 10.17796/1053-4628-40.6.456.
20. Fagade OO, Oginni FO. Intra-operative pain perception in tooth extraction--possible causes. Int Dent J. 2005;55:242-6.
21. Fan S, Chen WL, Pan CB, et al. Anesthetic efficacy of inferior alveolar nerve block plus buccal infiltration or periodontal ligament injections with articaine in patients with irreversible pulpitis in the mandibular first molar. Oral Surg Oral Med Oral Pathol Oral Radiol Endod. 2009;108:e89-93. doi: 10.1016/j.tripleo.2009.06.012. a
22. Fan S, Chen WL, Yang ZH, Huang ZQ. Comparison of the efficiencies of permanent maxillary tooth removal performed with single buccal infiltration versus routine buccal and palatal injection. Oral Surg Oral Med Oral Pathol Oral Radiol Endod. 2009;107:359-63. doi: 10.1016/j.tripleo.2008.08.025. b
23. Forloine A, Drum M, Reader A, Nusstein J, Beck M. A prospective, randomized, double-blind comparison of the anesthetic efficacy of two percent lidocaine with 1:100,000 epinephrine and three percent mepivacaine in the maxillary high tuberosity second division nerve block. J Endod. 2010;36:1770-7. doi: 10.1016/j.joen.2010.08.014.
24. Gibson RS, Allen K, Hutfless S, Beiraghi S. The wand vs. traditional injection: A comparison of pain related behaviors. Pediatr Dent. 2000;22:458-62.
25. Goodell GG, Gallagher FJ, Nicoll BK. Comparison of a controlled injection pressure system with a conventional technique. Oral Surg Oral Med Oral Pathol Oral Radiol Endod. 2000;90:88-94. doi: S1079-2104(00)13901-0.
26. Gregorio LV, Giglio FP, Sakai VT, et al. A comparison of the clinical anesthetic efficacy of 4% articaine and 0.5% bupivacaine (both with 1:200,000 epinephrine) for lower third molar removal. Oral Surg Oral Med Oral Pathol Oral Radiol Endod. 2008;106:19-28. doi: 10.1016/j.tripleo.2007.11.024.
27. Guglielmo A, Drum M, Reader A, Nusstein J. Anesthetic efficacy of a combination palatal and buccal infiltration of the maxillary first molar. J Endod. 2011;37:460-2. doi: 10.1016/j.joen.2011.01.005.
28. Haghgoo R, Taleghani F. Comparison of periodontal ligament injection and inferior alveolar nerve block in mandibular primary molars pulpotomy: A randomized control trial. J Int Oral Health. 2015;7:11-4.
29. Hoef N, Amerongen E. Influence of local anaesthesia on the quality of class II glass ionomer restorations. Int J Paediatr Dent. 2007;17:239-47. doi: 10.1111/j.1365-263X.2007.00818.x.
30. Hung PC, Chang HH, Yang PJ, Kuo YS, Lan WH, Lin CP. Comparison of the gow-gates mandibular block and inferior alveolar nerve block using a standardized protocol. J Formos Med Assoc. 2006;105:139-46. doi: S0929-6646(09)60335-1.
31. Isiordia-Espinoza MA, Orozco-Solis M, Tobí¬as-Azúa FJ, Méndez-Gutiérrez EP. Submucous tramadol increases the anesthetic efficacy of mepivacaine with epinephrine in inferior alveolar nerve block. Br J Oral Maxillofac Surg. 2012;50:157-60. doi: 10.1016/j.bjoms.2011.02.010.
32. Janjua OS, Luqman U, Ibrahim MW, Shah I. Transpapillary versus palatal injection technique for maxillary tooth extractions. J Coll Physicians Surg Pak. 2012;22:143-6. doi: 02.2012/JCPSP.143146.
33. Jing Q, Wan K, Wang X-, Ma L. Effectiveness and safety of computer-controlled periodontal ligament injection system in endodontic access to the mandibular posterior teeth. Chinese Medical Sciences Journal. 2014;29:23-7. Accessed 24 November 2016. doi: 10.1016/S1001-9294(14)60019-5.
34. Joyce AP, Donnelly JC. Evaluation of the effectiveness and comfort of incisive nerve anesthesia given inside or outside the mental foramen. J Endod. 1993;19:409-11. doi: 10.1016/S0099-2399(06)81506-2.
35. Kämmerer PW, Palarie V, Daubländer M, et al. Comparison of 4% articaine with epinephrine (1:100,000) and without epinephrine in inferior alveolar block for tooth extraction: Double-blind randomized clinical trial of anesthetic efficacy. Oral Surg Oral Med Oral Pathol Oral Radiol. 2012;113:495-9. doi: 10.1016/j.tripleo.2011.04.037.
36. Kämmerer PW, Kramer N, Esch J, et al. Epinephrine-reduced articaine solution (1:400,000) in paediatric dentistry: A multicentre non-interventional clinical trial. Eur Arch Paediatr Dent. 2013;14:89-95. doi: 10.1007/s40368-013-0024-9.
37. Kanaa MD, Whitworth JM, Meechan JG. A prospective randomized trial of different supplementary local anesthetic techniques after failure of inferior alveolar nerve block in patients with irreversible pulpitis in mandibular teeth. J Endod. 2012;38:421-5. doi: 10.1016/j.joen.2011.12.006.
38. Kandiah P, Tahmassebi JF. Comparing the onset of maxillary infiltration local anaesthesia and pain experience using the conventional technique vs. the wand in children. Br Dent J. 2012;213:E15. doi: 10.1038/sj.bdj.2012.988.
39. Klein U, Hunzeker C, Hutfless S, Galloway A. Quality of anesthesia for the maxillary primary anterior segment in pediatric patients: Comparison of the P-ASA nerve block using CompuMed delivery system vs traditional supraperiosteal injections. J Dent Child (Chic). 2005;72:119-25.
40. Kohler BR, Castellon L, Laissle G. Gow-gates technique: A pilot study for extraction procedures with clinical evaluation and review. Anesth Prog. 2008;55:2-8. doi: 10.2344/0003-3006(2008)55[2:GTAPSF]2.0.CO;2.
41. Kreimer T, Kiser R 2nd, Reader A, Nusstein J, Drum M, Beck M. Anesthetic efficacy of combinations of 0.5 mol/L mannitol and lidocaine with epinephrine for inferior alveolar nerve blocks in patients with symptomatic irreversible pulpitis. J Endod. 2012;38:598-603. doi: 10.1016/j.joen.2012.02.016.
42. Krzemiński TF, Gilowski L, Wiench R, Płocica I, Kondzielnik P, Sielańczyk A. Comparison of ropivacaine and articaine with epinephrine for infiltration anaesthesia in dentistry - a randomized study. Int Endod J. 2011;44:746-51. doi: 10.1111/j.1365-2591.2011.01881.x.
43. Kuscu OO, Akyuz S. Is it the injection device or the anxiety experienced that causes pain during dental local anaesthesia? Int J Paediatr Dent. 2008;18:139-45. doi: 10.1111/j.1365-263X.2007.00875.x.
44. Lima JL Jr, Dias-Ribeiro E, Ferreira-Rocha J, et al. Comparison of buccal infiltration of 4% articaine with 1 : 100,000 and 1 : 200,000 epinephrine for extraction of maxillary third molars with pericoronitis: A pilot study. Anesth Prog. 2013;60:42-5. doi: 10.2344/0003-3006-60.2.42.
45. Malamed SF, Tavana S, Falkel M. Faster onset and more comfortable injection with alkalinized 2% lidocaine with epinephrine 1:100,000. Compendium of continuing education in dentistry (Jamesburg, N.J.: 1995). 2013;34:10-20.
46. Maniglia-Ferreira C, Almeida-Gomes F, Carvalho-Sousa B, et al. Clinical evaluation of the use of three anesthetics in endodontics. Acta Odontol Latinoamericana : AOL. 2009;22:21-6.
47. Martínez-Rodríguez N, Barona-Dorado C, Martín-Arés M, Cortés-Bretón-Brinkman J, Martínez-González JM. Evaluation of the anaesthetic properties and tolerance of 1:100,000 articaine versus 1:100,000 lidocaine. A comparative study in surgery of the lower third molar. Med Oral Patol Oral Cir Bucal. 2012;17:e345-51.
48. Martinez Gonzalez JM, Benito Pena B, Fernandez Caliz F, San Hipolito Marin L, Penarrocha Diago M. A comparative study of direct mandibular nerve block and the akinosi technique. Med Oral. 2003;8:143-9. doi: 10488612.
49. Meechan JG, Cole B, Welbury RR. The influence of two different dental local anaesthetic solutions on the haemodynamic responses of children undergoing restorative dentistry: A randomised, single-blind, split-mouth study. Br Dent J. 2001;190:502-4. doi: 10.1038/sj.bdj.4801015a.
50. Mellor AC, Dorman ML, Girdler NM. The use of an intra-oral injection of ketorolac in the treatment of irreversible pulpitis. Int Endod J. 2005;38:789-92; discussion 792-4. doi: 10.1111/j.1365-2591.2005.01015_1.x.
51. Mittal M, Kumar A, Srivastava D, Sharma P, Sharma S. Pain perception: Computerized versus traditional local anesthesia in pediatric patients. J Clin Pediatr Dent. 2015;39:470-4. doi: 10.17796/1053-4628-39.5.470.
52. Odabas ME, Cinar C, Deveci C, Alacam A. Comparison of the anesthetic efficacy of articaine and mepivacaine in pediatric patients: A randomized, double-blind study. Pediatr Dent. 2012;34:42-5.
53. Oulis CJ, Vadiakas GP, Vasilopoulou A. The effectiveness of mandibular infiltration compared to mandibular block anesthesia in treating primary molars in children. Pediatr Dent. 1996;18:301-5.
54. Palm AM, Kirkegaard U, Poulsen S. The wand versus traditional injection for mandibular nerve block in children and adolescents: Perceived pain and time of onset. Pediatr Dent. 2004;26:481-4.
55. Parirokh M, Satvati SA, Sharifi R, et al. Efficacy of combining a buccal infiltration with an inferior alveolar nerve block for mandibular molars with irreversible pulpitis. Oral Surg Oral Med Oral Pathol Oral Radiol Endod. 2010;109:468-73. doi: 10.1016/j.tripleo.2009.11.016.
56. Pereira LA, Groppo FC, Bergamaschi Cde C, et al. Articaine (4%) with epinephrine (1:100,000 or 1:200,000) in intraosseous injections in symptomatic irreversible pulpitis of mandibular molars: Anesthetic efficacy and cardiovascular effects. Oral Surg Oral Med Oral Pathol Oral Radiol. 2013;116:e85-91. doi: 10.1016/j.oooo.2011.10.045.
57. Poorni S, Veniashok B, Senthilkumar AD, Indira R, Ramachandran S. Anesthetic efficacy of four percent articaine for pulpal anesthesia by using inferior alveolar nerve block and buccal infiltration techniques in patients with irreversible pulpitis: A prospective randomized double-blind clinical trial. J Endod. 2011;37:1603-7. doi: 10.1016/j.joen.2011.09.009.
58. Ram D, Hermida LB, Peretz B. A comparison of warmed and room-temperature anesthetic for local anesthesia in children. Pediatr Dent. 2002;24:333-6.
59. Ram D, Kassirer J. Assessment of a palatal approach-anterior superior alveolar (P-ASA) nerve block with the wand in paediatric dental patients. Int J Paediatr Dent. 2006;16:348-51. doi: 10.1111/j.1365-263X.2006.00752.x. a
60. Ram D, Amir E. Comparison of articaine 4% and lidocaine 2% in paediatric dental patients. International Journal of Paediatric Dentistry. 2006;16:252-6. Accessed 24 November 2016. doi: 10.1111/j.1365-263X.2006.00745.x. b
61. Ran D, Peretz B. Assessing the pain reaction of children receiving periodontal ligament anesthesia using a computerized device (wand). J Clin Pediatr Dent. 2003;27:247-50.
62. Reitz J, Reader A, Nist R, Beck M, Meyers WJ. Anesthetic efficacy of a repeated intraosseous injection given 30 min following an inferior alveolar nerve block/intraosseous injection. Anesth Prog. 1998;45:143-9. a
63. Reitz J, Reader A, Nist R, Beck M, Meyers WJ. Anesthetic efficacy of the intraosseous injection of 0.9 mL of 2% lidocaine (1:100,000 epinephrine) to augment an inferior alveolar nerve block. Oral Surg Oral Med Oral Pathol Oral Radiol Endod. 1998;86:516-23. b
64. Replogle K, Reader A, Nist R, Beck M, Weaver J, Meyers WJ. Anesthetic efficacy of the intraosseous injection of 2% lidocaine (1:100,000 epinephrine) and 3% mepivacaine in mandibular first molars. Oral Surg Oral Med Oral Pathol Oral Radiol Endod. 1997;83:30-7. doi: S1079-2104(97)90087-1.
65. Rogers BS, Botero TM, McDonald NJ, Gardner RJ, Peters MC. Efficacy of articaine versus lidocaine as a supplemental buccal infiltration in mandibular molars with irreversible pulpitis: A prospective, randomized, double-blind study. J Endod. 2014;40:753-8.
66. Said YS, Stein JM, Marioth-Wirtz E. Evaluation of the anesthetic effect of epinephrine-free articaine and mepivacaine through quantitative sensory testing. Head & face medicine. 2015;11:2. doi: 10.1186/s13005-015-0061-1.
67. Salomon E, Mazzoleni S, Sivolella S, et al. Age limit for infiltration anaesthesia for the conservative treatment of mandibular first molars. A clinical study on a paediatric population. European Journal of Paediatric Dentistry. 2012;13:259-62. Accessed 24 November 2016.
68. Sampaio RM, Carnaval TG, Lanfredi CB, Horliana AC, Rocha RG, Tortamano IP. Comparison of the anesthetic efficacy between bupivacaine and lidocaine in patients with irreversible pulpitis of mandibular molar. J Endod. 2012;38:594-7. doi: 10.1016/j.joen.2012.01.008.
69. Sandeep V, Kumar M, Jyostna P, Duggi V. Evaluation of 2-stage injection technique in children. Anesth Prog. 2016;63:3-7. doi: 10.2344/0003-3006-63.1.3.
70. Satish SV, Shetty KP, Kilaru K, Bhargavi P, Reddy ES, Bellutgi A. Comparative evaluation of the efficacy of 2% lidocaine containing 1:200,000 epinephrine with and without hyaluronidase (75 IU) in patients with irreversible pulpitis. J Endod. 2013;39:1116-8. Accessed 24 November 2016. doi: 10.1016/j.joen.2012.11.049.
71. Sharaf AA. Evaluation of mandibular infiltration versus block anesthesia in pediatric dentistry. ASDC J Dent Child. 1997;64:276-81.
72. Srinivasan N, Kavitha M, Loganathan CS, Padmini G. Comparison of anesthetic efficacy of 4% articaine and 2% lidocaine for maxillary buccal infiltration in patients with irreversible pulpitis. Oral Surg Oral Med Oral Pathol Oral Radiol Endod. 2009;107:133-6. doi: 10.1016/j.tripleo.2008.09.002.
73. Srisurang S, Narit L, Prisana P. Clinical efficacy of lidocaine, mepivacaine, and articaine for local infiltration. J Investig Clin Dent. 2011;2:23-28. doi: 10.1111/j.2041-1626.2010.00035.x.
74. Thongkukiatkun W, Vongsavan K, Kraivaphan P, Rirattanapong P, Vongsavan N, Matthews B. Effects of the iontophoresis of lignocaine with epinephrine into exposed dentine on the sensitivity of the dentine in man. Arch Oral Biol. 2015;60:1098-103.
75. Tudeshchoie DG, Rozbahany NA, Hajiahmadi M, Jabarifar E. Comparison of the efficacy of two anesthetic techniques of mandibular primary first molar: A randomized clinical trial. Dent Res J (Isfahan). 2013;10:620-3.
76. Uckan S, Dayangac E, Araz K. Is permanent maxillary tooth removal without palatal injection possible? Oral Surg Oral Med Oral Pathol Oral Radiol Endod. 2006;102:733-5. doi: S1079-2104(05)01116-9.
77. Willett J, Reader A, Drum M, Nusstein J, Beck M. The anesthetic efficacy of diphenhydramine and the combination diphenhydramine/lidocaine for the inferior alveolar nerve block. J Endod. 2008;34:1446-50. doi: 10.1016/j.joen.2008.09.005.
78. Yassen GH. Evaluation of mandibular infiltration versus mandibular block anaesthesia in treating primary canines in children. Int J Paediatr Dent. 2010;20:43-9. doi: 10.1111/j.1365-263X.2009.01023.x.
79. Yilmaz Y, Eyuboglu O, Keles S. Comparison of the efficacy of articaine and prilocaine local anaesthesia for pulpotomy of maxillary and mandibular primary molars. Eur J Paediatr Dent. 2011;12:117-22.
80. Zarei M, Ghoddusi J, Sharifi E, Forghani M, Afkhami F, Marouzi P. Comparison of the anaesthetic efficacy of and heart rate changes after periodontal ligament or intraosseous X-tip injection in mandibular molars: A randomized controlled clinical trial. Int Endod J. 2012;45:921-6. doi: 10.1111/j.1365-2591.2012.02050.x.
